# Male partners’ perceptions of maternal near miss obstetric morbidity experienced by their spouses

**DOI:** 10.1186/s12978-015-0011-1

**Published:** 2015-03-24

**Authors:** Scovia N Mbalinda, Annettee Nakimuli, Sarah Nakubulwa, Othman Kakaire, Michael O Osinde, Nelson Kakande, Dan K Kaye

**Affiliations:** Department of Nursing, School of Health Sciences, College of Health Sciences, Makerere University, P.O. Box 7072, Kampala, Uganda; Department of Obstetrics and Gynecology, School of Medicine, College of Health Sciences, Makerere University, P.O. Box 7072, Kampala, Uganda; Department of Obstetrics and Gynecology, Jinja Regional Hospital, Jinja, Uganda; Clinical, Operations and Health Services Research Program, Joint Clinical Research Centre, P. O. Box 10005, Kampala, Uganda

## Abstract

**Background:**

Severe obstetric complications have potential negative impact on the family and household of the survivors, with potential negative effects during (and in the aftermath of) the traumatic obstetric events. The objective was to gain deeper understanding of how severe obstetric complications are perceived by male partners, and their impact on the livelihoods of the family and community.

**Methods:**

Data was collected through 25 in-depth narrative interviews with male partners of women with severe obstetric morbidity. The interviews occurred 4-12 months after the traumatic childbirth events. To gain a deeper understanding of the meanings and spouses attach to the experiences, we employed the notions of social capital and resilience.

**Results:**

Male partners’ perceptions and experiences were mostly characterized by losses, dreams and dilemmas, disempowerment and alienation, seclusion and self isolation or reliance on the social networks. During the aftermath of the events, there was disruption of the livelihoods of the partners and the whole family.

**Conclusion:**

While a maternal near miss obstetric event might appear as a positive outcome for the survivors, partners and caregivers of women who experience severe obstetric morbidity are deeply affected by the experiences of this life-threatening episode.

## Background

Obstetric near miss refer to women who narrowly survive death from severe obstetric complications and conceptually, they represent a point on a continuum between extremes where pregnancy or childbirth event is uncomplicated, complicated, life-threatening or fatal [1-3]. Such maternal near miss is commonly associated with obstetric complications such as unsafe abortions, eclampsia, obstetric hemorrhage, obstructed labor and sepsis [1-3]. Maternal near miss obstetric events have profound physical, financial and social consequences among poor households [4-7], acting as causes or catalysts of vulnerability [8,9]. In the aftermath of the obstetric event, a vicious cycle of poverty, morbidity, disability and loss of livelihood might ensue for the survivors, whereby high healthcare costs, morbidity and loss of household productivity exacerbate each other [4-9]. Indeed, for many families in developing countries, maternal near miss morbidity is associated enduring ill health, disability, prohibitive household expenditures and loss of economic productivity [10-14].

Social capital refers to the collective value of all “social networks” (who people know, interpersonal trust and norms, which act as resources for individuals and facilitate cooperation and collective action) and the inclinations that arise from these networks to do things for each other (“norms of reciprocity”) and to support each other [15-18]. The central premise of social capital is that social networks have value and specific benefits that encompass trust, connectedness, reciprocity, information, and cooperation [15,18]. Vulnerability is defined as exposure to risk of adverse health or health demands in situations of inadequate social and economic resources needed to mitigate subsequent social and economic consequences [16]. Resilience’ refers to a positive outcome in the context of risk (factors known to be associated with negative outcomes) or quick recovery from illness or hardship, and relates to primary survival values or recovery of assets or livelihoods [16,17].

There is limited published research that explores the male partners’ experiences and perceptions of f traumatic childbirth associated with near-miss morbidity among survivors. In rural Uganda, research showed that men play an active role in birth preparedness and complication readiness, by providing essential material, financial and emotional support to women with obstetric complications [19]. A qualitative study on men’s experiences of maternal near miss complications [20] showed that while men were eager to play an active role in supporting their partners/wives/spouses during this time, they experience personal, relationship, family and community factors as barriers to their involvement [21,22]. In addition, there are barriers from an unwelcoming, intimidating and unsupportive health system. In developed country contexts, similar findings were noted [21,22], where some men felt helpless distressed or confused during their partners’ severe obstetric illness. Indeed, some felt disoriented or distressed and perceived themselves as failures in the aftermath of their spouse’s traumatic childbirth experience [23-26]. The objective was to explore and gain a deeper understanding of how sequelae of severe obstetric complications are perceived by male partners of survivors of maternal near miss morbidity.

## Methods

### Setting and participants

The study was conducted from April 2013 to March 2014 in three districts of rural Uganda. Data was collected through 25 in-depth narrative interviews with male partners of women admitted earlier with severe obstetric morbidity. Table 1 shows the brief socio-demographic profile of the participants.

**Table 1:** **Socio-demographic profiles of the participants**

**Age**	**Number of children**	**Education level**	**Employment status**
22	1	Primary	Formally employed
25	2	Primary	Informal employment
25	2	University	Informal employment
26	3	Primary	Informal employment
27	2	Secondary	Formally employed s
29	2	Primary	Informal employment
29	5	University	Informal employment
29	3	Secondary	Unemployed
30	7	Secondary	Informal employment
28	3	University	Formally employed
31	4	Secondary	Unemployed
32	3	Secondary	Formally employed
34	4	Secondary	Informal employment
36	4	Secondary	Unemployed
35	5	Secondary	Formally employed
38	7	Primary	Formally employed
38	8	Primary	Informal employment
41	1	University	Informal employment
41	6	Primary	Formally employed
44	2	Secondary	Formally employed
44	2	Primary	Unemployed
44	3	Primary	Formally employed
46	2	Secondary	Formally employed
47	2	Secondary	Formally employed
48	9	University	Formally employed

### Data collection

The study design was a phenomenological study on coping with acute healthcare crises among male partners of women who experienced obstetric near miss events 4-12 months earlier. These participants were identified through their spouses, who were recruited earlier in a study on maternal near miss [8,9]. All participants gave verbal informed consent and were assured that the information given was confidential, that they were not obliged to join the study, and that their views would be anonymous. All the participants were interviewed on two occasions. The second interview was arranged 3-6 months after the first interview. This design was found suitable since it enables individuals to analyze phenomena, events or experiences as they appear to them, and enables assessment of how the participants reflected on their own experiences and the meanings they attached to them. Participants were selected by maximum variation sampling to represent different age groups, education level, socio-economic status and relationship type. We used the notion of social capital, vulnerability and resilience to explore partners’ experiences and perceptions over the period of their spouses’ recovery from severe obstetric morbidity. Interviews were conducted in English and Luganda, a widely spoken Bantu language. The proceedings were tape recorded. Interviews lasted for 30-50 minutes. Hand written notes were taken during the interviews and later expanded into transcripts. At the end of each interview, the key points were summarized to the participant in order to verify the data.

### Data analysis

Thematic analysis was conducted from transcriptions of the in depth-interviews. The transcripts were read and phrases of text in which there were words with similar meanings were grouped into categories. Similar categories were aggregated into themes and sub-themes. The findings contain direct quotes from participants and the narrations are reported as they were spoken by participants to maintain the meaning. A theme was identified as a consistent pattern found in the information that described or interpreted aspects of the phenomenon. Easy Text (EZ) software was used for data retrieval during analysis.

### Ethical considerations

This research was part of a post-doctoral research project of the first author (DKK) on *Evaluation and surveillance of the impact of maternal and neonatal near-miss morbidity on the health of mothers and infants*. Ethical approval to conduct the study was obtained from the Ethics and research committees of Mulago hospital, the School of Medicine, Makerere University College of Health Sciences and Uganda National Council for Science and Technology. All participants gave verbal informed consent to be interviewed and for proceedings to be recorded.

## Results

### Losses, dreams and dilemmas

The male partners reported multiple losses related to the traumatic childbirth. This included intense fears and worries, financial loss, death of newborns (stillbirths or neonatal deaths), and loss of time where caregivers and male partners spent time in hospital during the patients’ hospitalization. One man whose wife had recurrent miscarriages had this to report:

*“She had lost two previous pregnancies. So when this happened, I lost hope.”*

Operative delivery was also considered a loss, as exemplified by one male partner of a women who had to undergo a repeat emergency caesarean section:

*“I was very scared. I was afraid to the extent that I was asking myself what could happen. I feared the operation, for her, for the baby and what might happen…”.*

Since many women experienced prolonged morbidity, not knowing what caused the illness and or what would happen in future was the most difficult part of the partners’ experience, as noted by a male partner of a woman who suffered eclampsia:

*“She was very sick for many days. I was not sure whether she would survive, though I remained prayerful and optimistic. I am still worried. What was this illness? What causes it? Does it have a cure? Is there a way to prevent it?. Will it recur in future?”*

Anticipation and expectations predisposed to poor judgment and distress, as described by one man:

*“I thought she would have a normal birth. Even when they advised her to go to hospital before onset of labor, I dissuaded her from doing so. I wish we accepted the advice.”*

### Mixed expectations, mixed experiences

Many men had often illogical or unrealistic expectations. Most preferred to have a natural childbirth, as this was viewed as a symbol of a ‘normal’ woman. Advice from others (older sisters, in-laws, friends with children, neighbors or workmates) was either gratefully appreciated or was found to be distressing or unhelpful. *Male partners’ experiences in hospital were characterized by powerlessness and exclusion. They wanted to be involved in the decision-making for the patient’s healthcare, but often were ignored or by healthcare providers:*

*“I wanted to be with her. But they do not accept your pleas to be around. Healthcare providers did not communicate to me or involve me in critical decisions. Much as I trusted them, I was powerless.”*

For some men, the experience of taking the newborn to the neonatal care unit, while the mother was also hospitalized and very sick in a different unit, was the most distressing part of the childbirth experience. The men reported worry about how the baby would survive without breastfeeding. There were also fears that the baby may be stolen or interchanged with another baby. In addition, there was the double challenge of suddenly having two very sick patients-a newborn baby and her mother. This was associated with mixed emotions: joy regarding the birth of the baby and intense sadness and fear that the birth may lead to death of the mother, or that the mother will be severely incapacitated in future:.

*“Why should someone die while giving birth to life? I was very worried. I could not go to work. I suddenly realized I did not know how to look after a small baby, and had a lot to learn in a few days.”*

The men greatly appreciated efforts by some healthcare providers to communicate to them about their patients’ welfare, much as it was difficult to explain the nature of some conditions or their cause. For instance, for some conditions like eclampsia, the medical cause is unknown, but some doctors made attempts to explain the basics of what the problem was and how they were going to manage it. To some men, this made a big difference. Therefore how well nurses and doctors communicated during or after the emergency was critical to the men’s ability to cope with the traumatic experience.

*“They were very helpful especially when they explained to me the basics of the condition where my wife had high blood pressure and fits. I think they made a good attempt trying to explain it in a way that a non-medical person can understand.”*

In contrast, some men felt that they did not get adequate communication about their patients, particularly regarding the prognosis and implications of the childbirth complications. Some men felt the healthcare providers were aloof, arrogant or hostile. While they could appreciate how difficult the job of managing so many patients was, they felt they had a right to ask or to be informed about what was going on.

*“It was a terrible experience. I thought I have a right to know. I thought they were very rude. They would at times reply using medical terminology and jargon.”*

### Invaluable support from the social networks

Many men described invaluable support from their colleagues at work, friends, family members and relatives during the time of the obstetric emergency and its aftermath. All the male partners reported acts of support and encouragement from the family and community, without which the aftermath would have been difficult. This is exemplified by one man:

*“I feel guilty and feel I am a failure. I could not save my young family. I feel I have let them down.”*

Factors that men perceived to improve ability to cope include being positive, optimism, and ability to regulate emotions (not to worry so much about the event), particularly knowing that it could have been worse that it was. The social network was of great help ensuring that the patients and their families could cope.

*“The members of the Mothers’ Union were very helpful. They emphasize that I should always be hopeful and thankful. Even the members of the church come here every week. So you feel a sense of belonging. Of course they also give financial support..”*

### Seclusion and self isolation

Some men tried to seek explanations from health workers about what the future had for them and their partners, but were left disappointed. For instance, few had explanations that surviving a ruptured uterus meant that the women might never conceive again, let alone remain like a normal woman, as they had to undergo an emergency hysterectomy where the uterus was removed. Other men who looked for support found it hard to get, and never got any acknowledgement of their distress from health workers. One man who persistently sought an explanation on his wife’s prognosis after a urinary fistula that followed a traumatic birth, and with a baby cerebral palsy, was devastated on finding the truth, and had this to say:

*“The doctors were unhelpful. They just said I was very lucky that both my wife and the baby were alive, as other men lost both. But at 7 months the baby cannot sit without support, and always gets fits. The mother is practically in hospital all the time.”*

Some men reported frequent flashbacks of the traumatic obstetric events, and could not take their minds away from their partners’ suffering. The distress was compounded by increased expenses through having to purchase medication, meet costs of hospital visits or where readmission to hospital occurred. Others found dealing with their trauma very isolating, and suffered insomnia and loneliness as it was hard to talk to family and friends.

*“It is very disturbing. My wife cannot stay without a pad. She always has a bad smell around her. Since she cannot go to the public in her condition, I also avoid going to public places.”*

Additionally, some men reported feeling persistent emptiness, with reduced interest in work, reduced ability in their performance at work, absenteeism, intense sadness and feelings of worthlessness.

## Discussion

The study shows that traumatic childbirth events provoke intense anxiety and fear in the male partners and have long-term consequences for them and their families. While healthcare providers might view a maternal near miss as an event with a positive outcome, male partners of the survivors perceive the maternal near miss as a protracted process with potential for negative outcomes for close family members. During hospitalization, support and communication which are highly valued are often lacking. The aftermath experiences and perceptions provide insight into the lasting effects of their partners’ traumatic childbirth experiences. The findings highlight the need to provide holistic care to families of survivors of maternal near miss, as in the current management paradigm, male partners feel side-lined and excluded.

The findings are in agreement with previous research on men’s experiences of obstetric emergencies [27-30], which underscore themes of alienation, disempowerment, information deprivation, involuntary separation and exclusion from partners, anxiety about their newborns and intricacies in caring for a fragile newborn at the time when the partner was undergoing recovery. The findings suggest that whereas the men interviewed were eager and willing to support the women in the time of the obstetric emergency and during the stormy recovery, some feel abandoned by their friends. They are dismayed by inadequate communication from healthcare providers regarding the patients’ condition. Additionally, the men find that their practical and emotional needs were met mainly by their social network rather than by healthcare professionals.

The partners’ alienation was at times self-inflicted, as some men became withdrawn from their social networks. Jessop and Fox [31], in their exploration of counselors’ experiences of providing counseling support for fathers who experienced traumatic birth, found that fathers were often reluctant to seek help and support, as (seeking help) was in contradiction to societal expectations. The findings indicate that some men experience “birth trauma” [32-35], whereby their mental health is affected by the events of the traumatic birth experienced by spouses. The findings that the social network plays a crucial role in men’s adjustment to the aftermath of traumatic obstetric events underscores the importance of the social network in men’s resilience and vulnerability to traumatic obstetric events [36-38]. Knowing there are others who feel the same way as you do or sharing those experiences with others who care can be powerful in helping you to deal with this kind of trauma.

In the context of this study, vulnerability refers to the prospect of the male partners experiencing physical or psychologicaly harm from the events or the traumatic births experienced by their spouses. Likewise, loss is a process rather than an event [5], Loss involves disruption of the “familiar forms and patterns of life” [5], whereby some individuals cope well and others become more vulnerable [5-7]. This conceptualization is in agreement with the description by Morrone *et al* [39] who define vulnerability as situations where individuals, households or communities subjected or exposed to potential harm from one or more risks, are unable foresee, endure, mitigate or recover from the harm resulting from such adverse shocks. Yet other individuals are able to endure and even overcome adversity, thus showing resilience [39].

Our study design could have introduced bias in the participant selection. Secondly, the participants were aware that researchers were healthcare providers. However, the interviews with male partners took place several months after the childbirth events, when the spouses were not undergoing healthcare. In addition, the interviews were conducted away from a healthcare facility. Furthermore, interviews were conducted on more than one occasion to assess whether there were any inconsistencies in male partners’ perspectives.

The findings highlight several operational principles that underlie resilience building for male partners of women survivors of traumatic obstetric events [40,41]: Firstly, social systems are inter-related, and though different types of triggers for vulnerability (referred to as shocks) may exist, they are interconnected and inter-related, leading to overlapping risks. Therefore response to these shocks requires adaptations at different spatial and temporal levels of the response system (individual, household or community levels) as well as addressing the underlying vulnerabilities [5-7,15,39-41]. Secondly, there is need to focus on “adaptive capacities”, that is on the ability of individuals in a community to deal with shocks based on different levels of exposure (magnitude of shocks and subsequent levels of stress) and degree of sensitivity (the extent to which actors or the whole systems are affected) [36-41]. Strategies may include increasing male participation in community activities, so as to strengthen their social networks as well as their “connectedness” to the social networks. In addition, the findings have implications for providing holistic care and addressing the mental health of male partners of maternal near miss survivors. Male partners play a critical role in caring for and supporting women and newborns during the postnatal period, which in turn impacts on long-tem health, coping ability and postnatal adjustment of the mothers [42-45] and health of the infant [46-50]. In contexts where fathers are more involved during labor, the women’s postnatal health is better [29], breastfeeding rates are higher and rates of child survival are increased [50]. Therefore, it is critical that the mental health of male partners of maternal near miss survivors is considered as part of family-centred care.
